# LASSO-based nomogram to predict early treatment failure in advanced hepatocellular carcinoma treated with SBRT, lenvatinib, and tislelizumab

**DOI:** 10.3389/fonc.2026.1905052

**Published:** 2026-10-07

**Authors:** Yingchun Zhao, Suming Wang, Li He, Xiaoqian Shen, Miaomiao Zhang, Sujing Zhang

**Affiliations:** Department of Oncology, The First Hospital of Hebei Medical University, Shijiazhuang, Hebei, China

**Keywords:** ALBI score, early treatment failure, hepatocellular carcinoma, LASSO regression, lenvatinib, nomogram, stereotactic body radiotherapy, systemic immune-inflammation index

## Abstract

**Background:**

Stereotactic body radiotherapy (SBRT) combined with lenvatinib and tislelizumab has shown potential activity in advanced hepatocellular carcinoma (HCC), but early treatment failure (ETF) remains common. We developed an exploratory model to estimate ETF, defined as radiologically confirmed progressive disease (PD) or all-cause death within 6 months after treatment initiation.

**Methods:**

This single-center retrospective study included advanced HCC patients treated with the triple regimen from June 2021 to June 2024. Baseline clinical, imaging, and hematological variables were analyzed. LASSO regression with 10-fold cross-validation was used for feature selection. Restricted cubic splines assessed linearity, and λ.1se was examined as a sensitivity analysis. A multivariable logistic regression model was presented as a nomogram. Discrimination, calibration, and decision curve analysis (DCA) were evaluated, with 1000-resample bootstrap internal validation repeating the modeling pipeline.

**Results:**

Among 118 patients, 33 (27.9%) experienced ETF; 28 had radiologically confirmed PD and 5 died without prior documented PD. At λ.min, LASSO selected maximum tumor diameter, Vp4 portal vein tumor thrombosis (PVTT), baseline systemic immune-inflammation index (SII), ALBI score, and log10-transformed alpha-fetoprotein (AFP). Under λ.1se, four predictors were retained, with SII excluded. The final model had an apparent ROC-AUC of 0.903 and an optimism-corrected ROC-AUC of 0.866. Calibration showed acceptable agreement between predicted and observed risks. DCA suggested net benefit over an exploratory threshold range of approximately 0.10-0.50, which should not be interpreted as a validated treatment-switching threshold.

**Conclusion:**

The nomogram showed promising internal discrimination for estimating 6-month ETF risk in advanced HCC patients receiving SBRT, lenvatinib, and tislelizumab. Given the modest sample size and single-center design, the model remains exploratory and hypothesis-generating. External multicenter validation is required before clinical implementation or use for treatment selection.

## 1 Introduction

Hepatocellular carcinoma (HCC) remains a formidable global health challenge, currently ranking as the sixth most common neoplasm and the third leading cause of cancer-related mortality worldwide (1). The epidemiological landscape of HCC is particularly severe in the Asia-Pacific region, where chronic hepatitis B virus (HBV) infection serves as the predominant etiological driver (2). Owing to its insidious onset and rapid progression, the majority of HCC patients are diagnosed at advanced stages (Barcelona Clinic Liver Cancer [BCLC] stage C), thereby precluding the application of curative modalities such as surgical resection, liver transplantation, or local ablation (3). Over the past decade, the paradigm of systemic therapy for advanced HCC has undergone a substantial shift. The advent of molecular targeted therapies (MTTs), primarily multiple receptor tyrosine kinase inhibitors (TKIs) like sorafenib and lenvatinib, initially breached the therapeutic bottleneck (4). Subsequently, the introduction of immune checkpoint inhibitors (ICIs), particularly programmed cell death protein 1 (PD-1) and programmed death-ligand 1 (PD-L1) antibodies, has fundamentally reshaped the treatment algorithm (5, 6). Notably, the combination of lenvatinib and tislelizumab, a humanized IgG4 anti-PD-1 monoclonal antibody engineered to minimize Fc gamma receptor (FcγR) binding on macrophages, has exhibited promising antitumor activity and manageable safety profiles in unresectable HCC, primarily through the dual blockade of tumor angiogenesis and restoration of anti-tumor immune surveillance (7).

Despite these recent therapeutic advances, the objective response rates (ORR) achieved by dual tyrosine kinase inhibitor (TKI) and immune checkpoint inhibitor (ICI) combinations in unresectable hepatocellular carcinoma (HCC) generally plateau at approximately 30% to 40%. For instance, a phase Ib study of lenvatinib plus pembrolizumab reported an ORR of 36.0% by RECIST v1.1, leaving a substantial proportion of patients experiencing *de novo* resistance or rapidly progressive disease after initial therapy (8). The integration of stereotactic body radiotherapy (SBRT) is increasingly explored as a local intensification strategy, particularly for patients with portal vein tumor thrombosis (PVTT), bulky hepatic lesions, or refractory disease not amenable to transarterial chemoembolization (TACE) (9). Preclinical evidence suggests that SBRT may remodel the tumor immune microenvironment by releasing damage-associated molecular patterns (DAMPs) and normalizing tumor vasculature, thereby potentially augmenting the efficacy of concurrent ICIs (10, 11). Recent clinical series have investigated SBRT combined with lenvatinib and tislelizumab in advanced HCC, with promising preliminary efficacy signals in single-arm studies (12, 13).

However, the clinical implementation of this triple therapy is associated with important challenges that critically undermine its universal applicability. From a health economics perspective, the concurrent administration of SBRT, lenvatinib, and tislelizumab imposes substantial financial toxicity on patients and healthcare systems (14–17). Furthermore, triple therapy is associated with cumulative and occasionally overlapping toxicities, including immune-related adverse events (irAEs) (18), which can lead to treatment interruption and compromise clinical efficacy. Identifying patients who are likely to fail early is therefore of paramount importance to spare them from exposure to ineffective, costly, and potentially toxic interventions.

To date, there is a limited availability of specific and robust predictive tools tailored to forecast early treatment failure (ETF) risk in patients undergoing stereotactic body radiotherapy (SBRT) combined with lenvatinib and tislelizumab. A biomarker, the systemic immune-inflammation index (SII), and the albumin-bilirubin (ALBI) score have been correlated with overall survival in HCC patients receiving immunotherapy or targeted therapy (19–21). Other clinical features, including the extent of portal vein tumor thrombosis (Vp4 involvement of the main portal trunk), have consistently been associated with poor prognosis in patients with advanced HCC (22–25).

The construction of a multivariable predictive model necessitates rigorous statistical methodologies, particularly when dealing with relatively limited sample sizes inherent to novel therapeutic combinations. Traditional stepwise regression methods are susceptible to overfitting and multicollinearity (26), and they have been widely criticized for producing unstable variable subsets. The Least Absolute Shrinkage and Selection Operator (LASSO) regression is a regularized technique that imposes an L1 penalty, shrinking coefficients of less informative variables to zero while preserving informative predictors (27–29). This method may reduce, but does not eliminate, the risk of overfitting in datasets of modest size. When reported transparently in line with the TRIPOD statement (30), such models can provide a preliminary and reproducible framework for hypothesis generation, although external validation is mandatory before any clinical use.

Therefore, to address the clinical need, the present study aims to comprehensively analyze a real-world cohort of advanced HCC patients treated with SBRT, lenvatinib, and tislelizumab. By leveraging the LASSO approach with internal bootstrap validation, calibration, and decision-curve analyses in accordance with the TRIPOD statement (30), we developed and internally validated a nomogram for predicting ETF. The nomogram is positioned as an exploratory, hypothesis-generating tool, and we explicitly acknowledge that it does not currently provide evidence-based guidance for individual treatment selection. External validation in independent multicenter cohorts is required before any clinical implementation can be considered.

## 2 Methods

### 2.1 Study design and ethical considerations

This was a retrospective, single-center, observational cohort study conducted at our institution. The study period spanned from June 2021 to June 2024, ensuring adequate follow-up duration for all enrolled patients. The study protocol was approved by the Institutional Review Board/Ethics Committee of The First Hospital of Hebei Medical University (Approval No. 2021-KY-054) and was conducted in accordance with the Declaration of Helsinki (31). Given the retrospective observational design and the use of de-identified data, the requirement for written informed consent was waived by the ethics committee. This report follows the TRIPOD statement for prediction model development (30); a completed TRIPOD checklist is provided as a separate supplementary file.

### 2.2 Patient selection

We systematically screened the electronic medical records of patients diagnosed with advanced HCC who received SBRT, lenvatinib, and tislelizumab. The inclusion criteria were: (1) definitive diagnosis of HCC confirmed by histopathology or non-invasive dynamic imaging criteria endorsed by the American Association for the Study of Liver Diseases guidelines (32); (2) BCLC stage C, characterized by macroscopic vascular invasion or extrahepatic metastasis; (3) ECOG performance status 0 or 1; (4) Child-Pugh class A or well-compensated class B (score ≤7); (5) at least one measurable intrahepatic target lesion according to mRECIST (33); and (6) receipt of the triple combination as first-line systemic treatment. Exclusion criteria were: (1) previous systemic therapy for HCC (n=12); (2) concurrent other primary malignancy or severe autoimmune disease requiring systemic immunosuppression; (3) incomplete baseline clinical, laboratory, or radiological data required for the candidate-variable set (n=10); and (4) follow-up shorter than 6 months for non-death reasons without documented ETF (n=12). The final analytical cohort comprised 118 patients. The 33 ETF events yielded an event-per-variable ratio of 6.6 for the five-predictor final model; this limited event number was considered when interpreting model stability and generalizability. The study selection and modeling workflow are shown in **Figure 1**.

**Figure 1:**
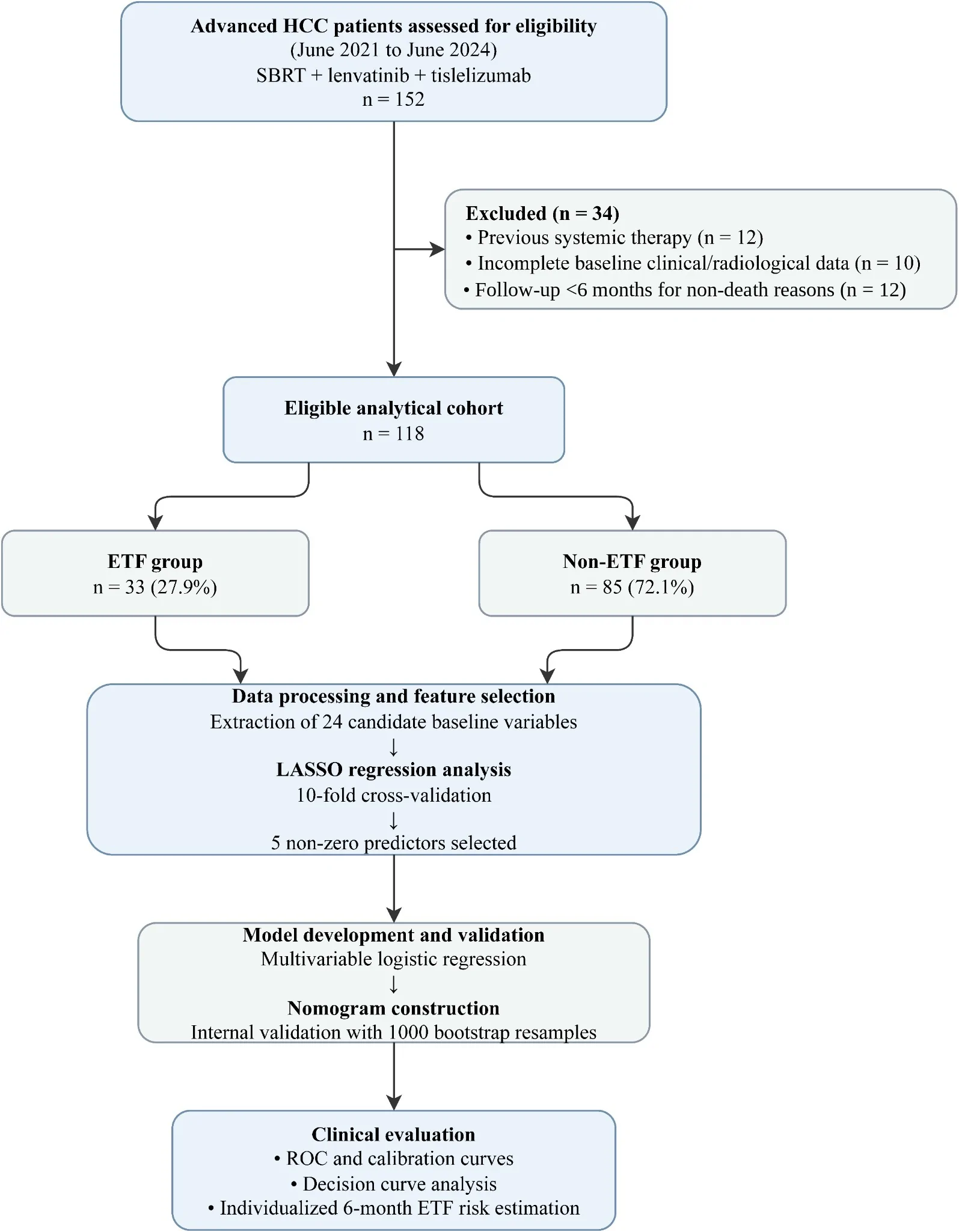
Study design and workflow. A total of 152 patients with advanced HCC who received SBRT, lenvatinib, and tislelizumab from June 2021 to June 2024 were assessed for eligibility. Thirty-four were excluded because of previous systemic therapy (n=12), incomplete baseline clinical/radiological data (n=10), or follow-up shorter than 6 months for non-death reasons (n=12). The final analytical cohort included 118 patients: 33 with ETF and 85 without ETF. The modeling workflow included 24 candidate baseline variables, LASSO feature selection, multivariable logistic regression, nomogram construction, and internal bootstrap validation.

### 2.3 Treatment protocol

The triple combination therapy was administered following a standardized institutional protocol. Lenvatinib was prescribed orally at a weight-based dosage: 12 mg once daily for patients weighing ≥60 kg and 8 mg once daily for patients weighing<60 kg. Tislelizumab was administered intravenously at 200 mg every three weeks (Q3W) until disease progression, intolerable toxicity, or patient withdrawal. Both agents were continued until one of the above criteria was met.

SBRT was delivered using the CyberKnife Robotic Radiosurgery System (Accuray Inc., Sunnyvale, CA, USA) with image guidance and respiratory motion management. The total dose ranged from 30 to 50 Gy in 3-5 fractions. BED10 and EQD2 were calculated using an α/β ratio of 10 Gy, and detailed dose-volume parameters are summarized in **Table 1**. The target volume encompassed the primary viable hepatic lesion and, when clinically indicated and technically feasible, macroscopic PVTT. Organ-at-risk constraints followed institutional practice, including attention to normal liver mean dose and V20 as well as maximum stomach and duodenal doses. The timing of SBRT relative to lenvatinib initiation, lenvatinib dose reductions or interruptions, tislelizumab delays, permanent treatment discontinuations, and grade ≥3 toxicities were recorded and are summarized descriptively in **Tables 1**, **2** because these treatment-level factors may influence early outcomes.

**Table 1:** SBRT treatment parameters and dose-volume metrics (n = 118).

SBRT parameter	Total (n = 118)	Non-ETF (n = 85)	ETF (n = 33)	P value
Total dose (Gy), n (%)				
30-39	25 (21.2)	17 (20.0)	8 (24.2)	0.654
40-49	69 (58.5)	50 (58.8)	19 (57.6)	
50	24 (20.3)	18 (21.2)	6 (18.2)	
Fractions, n (%)				
3	11 (9.3)	9 (10.6)	2 (6.1)	0.461
4	24 (20.3)	17 (20.0)	7 (21.2)	
5	83 (70.3)	59 (69.4)	24 (72.7)	
BED_10_ (Gy), median (IQR)	78.0 (72.0-100.0)	78.0 (72.0-100.0)	78.0 (72.0-100.0)	0.498
EQD2 (Gy), median (IQR)	66.7 (60.0-83.3)	66.7 (60.0-83.3)	66.7 (60.0-83.3)	0.498
GTV volume (cc), median (IQR)	146 (75-258)	128 (62-242)	178 (102-298)	0.067
PTV volume (cc), median (IQR)	262 (135-465)	230 (115-435)	320 (184-512)	0.054
Mean liver dose (Gy), median (IQR)	11.0 (9.0-13.0)	10.5 (8.8-12.6)	11.8 (9.6-13.8)	0.118
Liver V_20_ (%), median (IQR)	20.0 (16.0-25.0)	20.0 (15.5-24.5)	21.0 (17.0-26.0)	0.156
Stomach Dmax (Gy), median (IQR)	26.0 (22.5-29.0)	25.5 (22.0-28.5)	27.0 (23.5-29.5)	0.184
Duodenum Dmax (Gy), median (IQR)	24.0 (20.5-27.5)	23.5 (20.0-27.0)	25.0 (21.5-28.5)	0.151
PVTT included in RT target, n (%)	29/33 (87.9)	12/15 (80.0)	17/18 (94.4)	0.301
Days from SBRT start to Lenvatinib start, median (IQR)	7 (-3 to 14)	6 (-2 to 13)	9 (0 to 18)	0.082
Sequencing category, n (%)				0.491
Concurrent (within 3 days)	22 (18.6)	17 (20.0)	5 (15.2)	
Sandwich (4-14 days)	49 (41.5)	37 (43.5)	12 (36.4)	
Sequential (>14 days)	47 (39.8)	31 (36.5)	16 (48.5)	

SBRT was delivered using the CyberKnife robotic radiosurgery system. BED10 was calculated as D × (1 + d/(α/β)) with α/β = 10 Gy, and EQD2 as D × (d + α/β)/(2 + α/β). Dose-volume metrics and treatment timing are reported descriptively to characterize treatment heterogeneity; they were not used as candidate predictors in the primary LASSO model.

**Table 2:** Breakdown of early treatment failure events and treatment-related toxicity outcomes.

Outcome category	Total (n = 118)	Non-ETF (n = 85)	ETF (n = 33)	Note
ETF events - overall, n (%)	33 (27.9)	0	33 (100.0)	Composite endpoint within 180 days of treatment initiation
Radiologically confirmed PD by mRECIST, n (%)	28 (23.7)	0	28 (84.8)	Subcomponent of ETF endpoint
All-cause death without prior documented PD, n (%)	5 (4.2)	0	5 (15.2)	Subcomponent of ETF endpoint
Tumor progression as primary cause, n	2	0	2	
Hepatic decompensation (liver failure/hepatic encephalopathy), n	2	0	2	
Treatment-related toxicity, n	1	0	1	
Alive without documented progression at 6 months, n (%)	85 (72.0)	85 (100.0)	0	Patients followed for ≥180 days without PD or death
Time from treatment initiation to ETF event (days), median (IQR)			92 (61-138)	Among the 33 ETF cases
Treatment interruption or dose reduction, n (%)	37 (31.4)	23 (27.1)	14 (42.4)	Includes lenvatinib dose reduction, lenvatinib interruption ≥3 days, or tislelizumab dose delay
Permanent treatment discontinuation, n (%)	17 (14.4)	9 (10.6)	8 (24.2)	Any of SBRT, lenvatinib, or tislelizumab permanently discontinued
Due to hepatic decompensation, n	4	1	3	
Due to grade ≥3 hepatic toxicity, n	5	2	3	
Due to radiologically confirmed PD, n	5	4	1	
Due to other reasons, n	3	2	1	
Grade ≥3 adverse event (any cause, any type), n (%)	35 (29.7)	21 (24.7)	14 (42.4)	CTCAE v5.0
Hepatic (elevated transaminases or bilirubin), n	14	8	6	
Hematologic (neutropenia, thrombocytopenia, anemia), n	10	7	3	
Gastrointestinal (nausea, diarrhea, mucositis), n	6	4	2	
Other, n	5	2	3	

ETF was defined as radiologically confirmed progressive disease (PD) according to mRECIST or all-cause death within 180 days after treatment initiation, whichever occurred first. Permanent treatment discontinuation was defined as definitive cessation of lenvatinib and/or tislelizumab for any recorded reason; reasons are reported separately and sum to the overall count. Treatment interruption, dose reduction, or discontinuation due solely to toxicity did not constitute ETF unless PD or death occurred within 180 days.

### 2.4 Data collection and variable definitions

A comprehensive array of baseline variables was extracted within 7 days prior to the initiation of the triple therapy. The collected data encompassed three main domains: (1) Demographics and clinical characteristics including age, sex, etiology of liver disease (HBV, HCV, or other), ECOG PS, Child-Pugh class and score, and BCLC stage; (2) imaging parameters including maximum tumor diameter, tumor number (single vs. multiple), PVTT extent (categorized as none/Vp1-2/Vp3/Vp4), and extrahepatic spread (EHS); and (3) laboratory values and composite indices including white blood cell count, absolute neutrophil count, absolute lymphocyte count, hemoglobin, platelet count, albumin, total bilirubin, alanine aminotransferase, aspartate aminotransferase, alpha-fetoprotein (AFP), protein induced by vitamin K absence or antagonist-II (PIVKA-II), neutrophil-to-lymphocyte ratio (NLR), platelet-to-lymphocyte ratio (PLR), the Systemic Immune-Inflammation Index (SII = platelet count × neutrophil count/lymphocyte count) (34), and the Albumin-Bilirubin (ALBI = (log_10_ total bilirubin [μmol/L] × 0.66) − (albumin [g/L] × 0.085)) (35, 36). Continuous variables, including tumor diameter, ALBI score, and SII were modeled on their original scale; SII was scaled per 100-unit increment to facilitate clinical interpretation; AFP and PIVKA-II were log_10_-transformed before modeling due to skewed distributions and the raw values were retained in **Table 3** for clinical interpretability.

**Table 3:** Baseline characteristics of the study cohort (N = 118).

Characteristics	Total (n = 118)	Non-ETF (n = 85)	ETF (n = 33)	P value
Age (years), median (IQR)	58 (49-65)	57 (48-64)	61 (52-67)	0.214
Sex, n (%)				0.785
Male	103 (87.3)	75 (88.2)	28 (84.8)	
Female	15 (12.7)	10 (11.8)	5 (15.2)	
Etiology, n (%)				0.892
HBV	100 (84.7)	72 (84.7)	28 (84.8)	
HCV or others	18 (15.3)	13 (15.3)	5 (15.2)	
ECOG PS, n (%)				0.341
0	82 (69.5)	62 (72.9)	20 (60.6)	
1	36 (30.5)	23 (27.1)	13 (39.4)	
Child-Pugh class, n (%)				0.183
A	93 (78.8)	70 (82.4)	23 (69.7)	
B	25 (21.2)	15 (17.6)	10 (30.3)	
Maximum tumor diameter (cm), median (IQR)	8.5 (5.2-11.4)	7.2 (4.6-9.8)	10.8 (7.5-13.6)	<0.001
Tumor number, n (%)				0.452
Single	34 (28.8)	26 (30.6)	8 (24.2)	
Multiple	84 (71.2)	59 (69.4)	25 (75.8)	
Vp4 PVTT, n (%)				0.001
Absent	85 (72.0)	70 (82.4)	15 (45.5)	
Present	33 (28.0)	15 (17.6)	18 (54.5)	
Extrahepatic spread, n (%)				0.076
Absent	71 (60.2)	55 (64.7)	16 (48.5)	
Present	47 (39.8)	30 (35.3)	17 (51.5)	
Baseline SII, median (IQR)	560 (320-890)	450 (280-670)	820 (540-1250)	<0.001
ALBI score, median (IQR)	-2.52 (-2.81 to -2.15)	-2.65 (-2.88 to -2.35)	-2.18 (-2.45 to -1.90)	<0.001
Baseline AFP (ng/mL), median (IQR)	400 (35-4000)	210 (20-2500)	1800 (150-12000)	0.012
SBRT total dose (Gy), median (IQR)	40 (40-50)	40 (40-50)	40 (40-50)	0.512
SBRT BED_10_ (Gy), median (IQR)	78.0 (72.0-100.0)	78.0 (72.0-100.0)	78.0 (72.0-100.0)	0.498
SBRT EQD2 (Gy), median (IQR)	66.7 (60.0-83.3)	66.7 (60.0-83.3)	66.7 (60.0-83.3)	0.498
PVTT included in RT target (among Vp4+), n (%)	29/33 (87.9)	12/15 (80.0)	17/18 (94.4)	0.301
Lenvatinib dose reduction, n (%)	26 (22.0)	17 (20.0)	9 (27.3)	0.388
Lenvatinib interruption (days), median (IQR)	5 (0-12)	4 (0-10)	8 (0-18)	0.041
Tislelizumab dose delay, n (%)	14 (11.9)	9 (10.6)	5 (15.2)	0.482
Grade ≥3 adverse event (any cause), n (%)	35 (29.7)	21 (24.7)	14 (42.4)	0.057
Permanent treatment discontinuation, n (%)	17 (14.4)	9 (10.6)	8 (24.2)	0.060

ETF, early treatment failure; IQR, interquartile range; HBV, hepatitis B virus; HCV, hepatitis C virus; ECOG PS, Eastern Cooperative Oncology Group performance status; PVTT, portal vein tumor thrombosis; SII, systemic immune-inflammation index; ALBI, albumin-bilirubin; AFP, alpha-fetoprotein; SBRT, stereotactic body radiotherapy; BED, biologically effective dose; EQD2, equivalent dose in 2-Gy fractions; RT, radiotherapy. AFP and PIVKA-II were log_10_-transformed for LASSO regression because of their skewed distributions; raw values are presented for clinical interpretability. SII was retained on its original scale and scaled per 100-unit increase in modeling. Continuous variables were compared between ETF and non-ETF groups using the Mann-Whitney U test; categorical variables using the chi-square or Fisher exact test.

### 2.5 Follow-up and definition of early treatment failure (ETF)

Patients were closely monitored following the initiation of therapy. Follow-up assessments, including physical examinations, dynamic contrast-enhanced computed tomography (CT) or magnetic resonance imaging (MRI), and serum AFP measurements, were performed every 4-6 weeks during the first 6 months and every 8-12 weeks thereafter, until disease progression, death, or loss to follow-up.

ETF was defined as radiologically confirmed PD according to mRECIST (33) or all-cause death within 6 months (180 days) after treatment initiation. Patients without radiologically confirmed PD or death within 180 days were classified as non-ETF. Among the 33 ETF events, 28 (84.8%) were radiologically confirmed PD and 5 (15.2%) were deaths without prior documented PD; the recorded causes of death were tumor progression (n=2), hepatic decompensation (n=2), and treatment-related toxicity (n=1). Treatment interruption, dose reduction, or permanent discontinuation due solely to toxicity was not regarded as ETF when PD or death did not occur within 180 days; such patients were classified according to their documented 6-month radiological and survival status. The 6-month cutoff was a prespecified operational endpoint for this study and should not be interpreted as a universally accepted consensus definition of ETF in HCC.

### 2.6 Feature selection and statistical analysis

The statistical analysis was designed to develop an interpretable preliminary prediction model while recognizing the limited number of ETF events. Candidate variables were entered as continuous variables unless otherwise specified. Maximum tumor diameter, ALBI score, SII, and log10-transformed AFP and PIVKA-II were modeled continuously; Vp4 PVTT was modeled as a binary variable. SII was scaled per 100-unit increase. Continuous predictors were standardized internally during LASSO fitting so that variables measured on different scales received a comparable penalty; coefficients in the final unpenalized logistic regression model are reported on their original clinical scales (37, 38).

LASSO regression was used for feature selection (27, 39). The penalization parameter (λ) was determined by 10-fold cross-validation using binomial deviance. λ.min was used for the primary model because it minimized the cross-validated deviance. The more conservative λ.1se criterion was examined in a sensitivity analysis to assess whether the selected feature set remained stable under stronger penalization.

Because the sample size and event number were modest (n=118; 33 ETF events), overfitting could not be eliminated by penalization alone. Internal validation therefore used 1000 bootstrap resamples; within each resample, data standardization, LASSO feature selection, λ selection, and logistic-model refitting were repeated before estimating optimism in discrimination. The optimism-corrected ROC-AUC was calculated from the apparent ROC-AUC after subtracting mean bootstrap optimism. Bootstrap validation was used to quantify internal optimism and does not substitute for external validation.

Potential non-linearity of maximum tumor diameter, SII, ALBI score, and log10 AFP was assessed using restricted cubic splines with four knots at the 10th, 35th, 65th, and 90th percentiles. Linear and spline-augmented models were compared using likelihood-ratio tests. No statistically significant non-linear component was detected for any of the four predictors (all P>0.05; **Table 4**); linear terms were therefore retained to preserve parsimony in the 33-event dataset (40).

**Table 4:** Multivariable logistic regression analysis of predictors for early treatment failure selected by LASSO.

Variable	β coefficient	SE (β)	Odds ratio (OR)	95% confidence interval (CI)	P value
Intercept (logit scale)	-1.213	1.976	0.297	0.006-14.29	0.539
Maximum tumor diameter (per 1-cm increase)	0.154	0.071	1.17	1.01-1.34	0.030
Vp4 PVTT (present vs. absent)	1.294	0.574	3.65	1.18-11.25	0.024
Baseline SII (per 100 units increase)	0.166	0.069	1.18	1.03-1.35	0.016
ALBI score (continuous, per 1-unit increase)	2.018	0.726	7.53	1.81-31.21	0.005
Log_10_ AFP (continuous, per 1-unit increase)	0.727	0.246	2.07	1.28-3.35	0.003

Full prediction equation: logit(P) = −1.213 + 0.154 × maximum tumor diameter (cm) + 1.294 × Vp4 PVTT (1 = present, 0 = absent) + 0.165 × SII (per 100 units) + 2.018 × ALBI score + 0.727 × log_10_(AFP [ng/mL]). The probability of ETF is then P = 1 / (1 + exp(−logit(P))). Variables included in the final multivariable logistic regression model were those with non-zero coefficients in the LASSO regression. Coefficients are reported on the original (unstandardized) scale of each predictor. SE, standard error; OR, odds ratio; CI, confidence interval; PVTT, portal vein tumor thrombosis; SII, systemic immune-inflammation index; ALBI, albumin-bilirubin; AFP, alpha-fetoprotein.

All analyses were performed in R version 4.3.2 (R Foundation for Statistical Computing, Vienna, Austria). LASSO regression was performed with glmnet version 4.1-8; logistic modeling, nomogram construction, calibration, and bootstrap procedures used rms version 6.7-1; ResourceSelection version 0.3-6 was used for supporting model diagnostics. Decision curve analysis was implemented in R using the standard net-benefit formulation described by Vickers and colleagues (41, 42). Continuous variables were compared between ETF and non-ETF groups using the Mann-Whitney U test; categorical variables were compared using the chi-square or Fisher exact test, as appropriate. A two-sided P<0.05 was considered statistically significant.

### 2.7 Nomogram construction and validation

Following LASSO feature selection, variables retaining non-zero coefficients were incorporated into an unpenalized multivariable logistic regression model. The results were visually translated into a nomogram to provide clinicians with a practical bedside risk calculator (40). The full prediction model was specified as:


$$logit\left(P\right)\;=\;-1.213\;+\;0.154\;\times \;maximum\;tumor\;diameter\;\left(cm\right)\;+\;1.294\;\times \;Vp4\;PVTT\;\left(1\;=\;present,\;0\;=\;absent\right)\;+\;0.165\;\times \;SII\;\left(per\;100\;units\right)\;+\;2.018\;\times \;ALBI\;score\;+\;0.727\;\times \;{\text{log}}_{10}\left(AFP\;\left[ng/mL\right]\right)$$

The probability of ETF is then obtained by applying the inverse-logit transformation P = 1/(1 + exp(−logit(P))). The nomogram was constructed to allow bedside use of this equation.

Discrimination was summarized using the area under the receiver operating characteristic curve (ROC-AUC). For a binary logistic outcome, the concordance statistic is numerically equivalent to the ROC-AUC; ROC-AUC is used preferentially here for clarity. Internal validation used the 1000-iteration bootstrap procedure described above, and the optimism-corrected ROC-AUC is reported (43).

Calibration was assessed by plotting predicted ETF probabilities against observed ETF proportions across risk groups. The 45-degree line represents ideal calibration. Because this is a single-center development cohort, calibration findings are interpreted as internal performance only and require confirmation in an independent cohort.

Clinical utility was explored using decision curve analysis (DCA) (41, 42), which quantifies net benefit across threshold probabilities. The interval from 0.10 to 0.50 was displayed as an exploratory threshold range because the nomogram showed higher net benefit than the default strategies over this part of the curve in the present cohort. These thresholds represent preference-sensitive trade-offs between false-positive and false-negative classifications and should not be interpreted as validated cutoffs for withholding, changing, or de-escalating triple therapy.

## 3 Results

### 3.1 Patient characteristics and ETF incidence

Between June 2021 and June 2024, 152 patients with advanced HCC who received SBRT, lenvatinib, and tislelizumab were assessed for eligibility. Thirty-four were excluded: 12 because of previous systemic therapy, 10 because of incomplete baseline clinical or radiological data, and 12 because follow-up was shorter than 6 months for non-death reasons without documented ETF. The final analytical cohort comprised 118 patients, including 103 men and 15 women, with a median age of 58 years (IQR 49-65). Thirty-three patients (27.9%) experienced ETF. Of these, 28 had radiologically confirmed PD and 5 died without prior documented PD within 6 months. The corresponding recorded causes of death were tumor progression (n=2), hepatic decompensation (n=2), and treatment-related toxicity (n=1). Baseline characteristics are summarized in **Table 3**, and the ETF component and treatment-interruption outcomes are summarized in **Table 2**.

### 3.2 Feature selection via LASSO regression

A pool of 24 candidate baseline variables (**Table 3**) was entered into LASSO regression with 10-fold cross-validation. At λ.min = 0.046, five variables retained non-zero coefficients: maximum tumor diameter, Vp4 PVTT, baseline SII (per 100 units), ALBI score, and log10 AFP. The LASSO coefficient paths and 10-fold cross-validation results are shown in **Figure 2**. Restricted-cubic-spline analyses did not identify significant non-linearity for the four continuous selected predictors (**Table 4**). In the λ.1se sensitivity analysis, maximum tumor diameter, Vp4 PVTT, ALBI score, and log10 AFP were retained, whereas SII was excluded under the stronger penalty (**Table 2**). The small change in discrimination in the sensitivity model suggests that the core feature set was reasonably stable, while also indicating that SII selection was sensitive to the degree of penalization. Bootstrap selection stability is summarized in **Table 1**.

**Figure 2:**
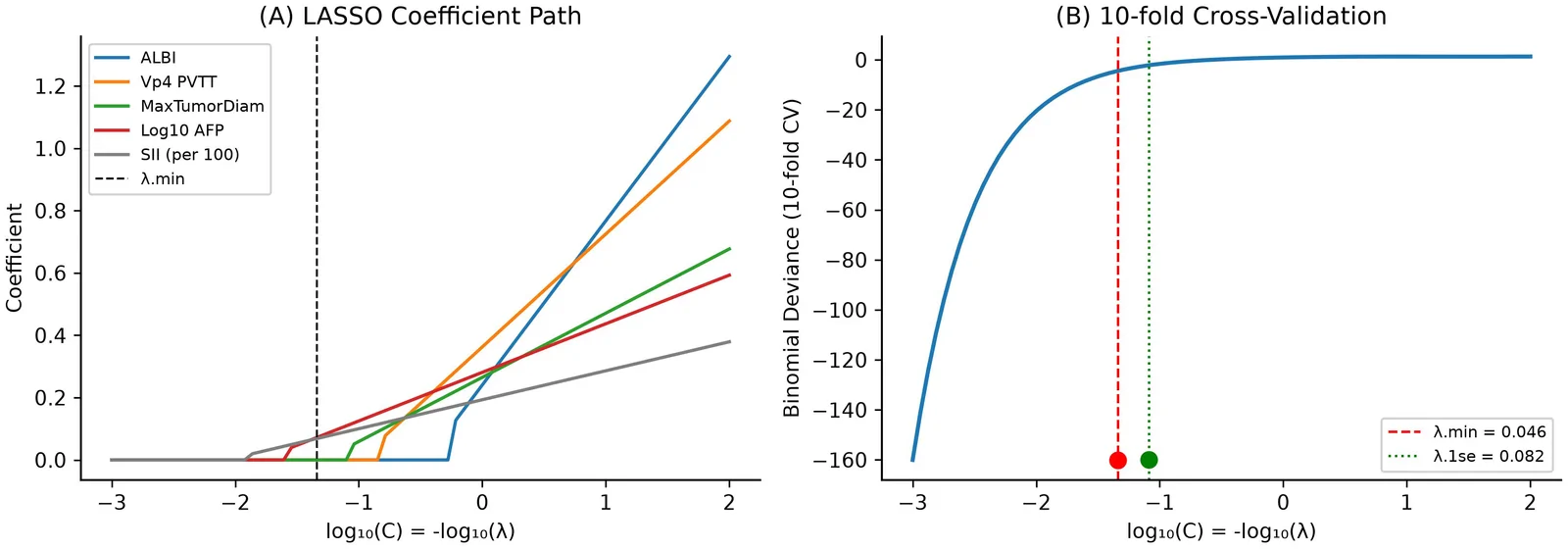
Feature selection using LASSO regression. **(A)** Coefficient profiles of the 24 candidate variables across the regularization path. **(B)** Ten-fold cross-validation curve used to determine the penalty parameter. The primary model used λ.min = 0.046 and retained five predictors: maximum tumor diameter, Vp4 PVTT, baseline SII, ALBI score, and log10 AFP. Under the more conservative λ.1se criterion, four predictors were retained and SII was excluded, indicating partial rather than complete stability of feature selection.

### 3.3 Development of the predictive nomogram

The five LASSO-selected features were entered into an unpenalized multivariable logistic regression model to develop the final prediction model. The model intercept was −1.213 (SE 1.976; P = 0.539 against zero) on the logit scale. The full prediction equation was:


$$logit\left(P\right)\;=\;-1.213\;+\;0.154\;\times \;maximum\;tumor\;diameter\;\left(cm\right)\;+\;1.294\;\times \;Vp4\;PVTT\;\left(1\;=\;present\right)\;+\;0.165\;\times \;SII\;\left(per\;100\;units\right)\;+\;2.018\;\times \;ALBI\;score\;+\;0.727\;\times \;{\text{log}}_{10}\left(AFP\;\left[ng/mL\right]\right)$$

All five variables were independently associated with ETF risk in the final model (**Table 4**). The strongest association was observed for ALBI score (OR 7.53, 95% CI 1.81-31.21; P = 0.005), followed by Vp4 PVTT (OR 3.65, 95% CI 1.18-11.25; P = 0.024) and log_10_ AFP (OR 2.07, 95% CI 1.28-3.35; P = 0.003). Maximum tumor diameter (per 1-cm increase) was associated with an OR of 1.17 (95% CI 1.01-1.34; P = 0.030), and SII (per 100-unit increase) with an OR of 1.18 (95% CI 1.03-1.35; P = 0.016). The nomogram for bedside risk calculation is presented in **Figure 3**.

**Figure 3:**
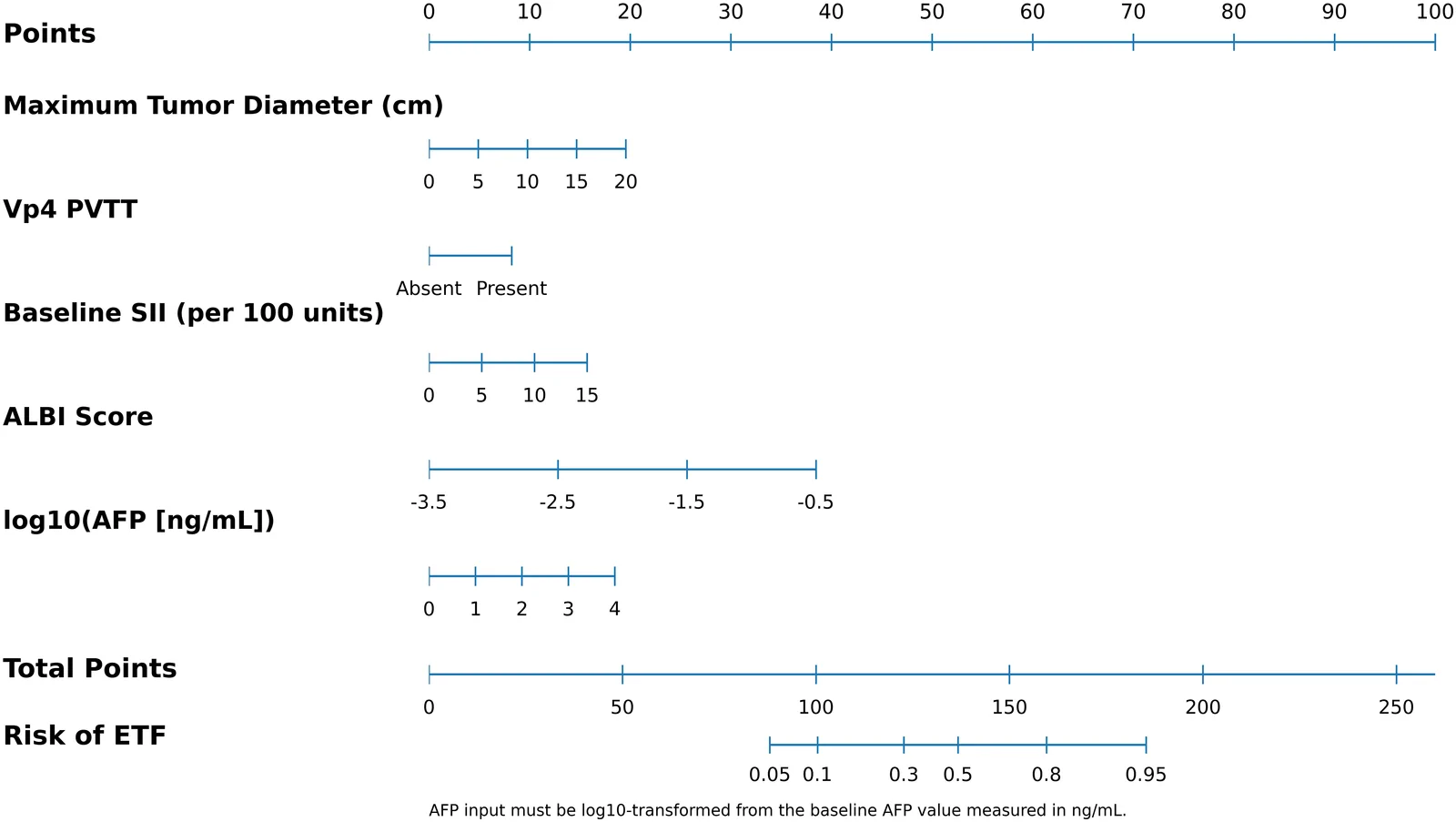
Nomogram for estimating 6-month early treatment failure. The final logistic model included maximum tumor diameter, Vp4 PVTT, baseline SII per 100 units, ALBI score, and log10(AFP [ng/mL]). Raw baseline AFP must first be transformed as log10(AFP in ng/mL); for example, 1000 ng/mL corresponds to a transformed value of 3. The full model equation is: logit(P) = −1.213 + 0.154 × maximum tumor diameter (cm) + 1.294 × Vp4 PVTT + 0.165 × SII (per 100 units) + 2.018 × ALBI score + 0.727 × log10(AFP [ng/mL]). The nomogram is intended for research risk estimation and not as a validated treatment-selection rule.

### 3.4 Internal validation and model performance

The final model showed an apparent ROC-AUC of 0.903 in the development cohort. After 1000-iteration bootstrap internal validation with repetition of the modeling pipeline within resamples, the optimism-corrected ROC-AUC was 0.866. These estimates indicate promising internal discrimination but should not be interpreted as evidence of external transportability. The calibration plot (**Figure 4**) showed generally acceptable agreement between predicted and observed ETF risks in the development cohort.

**Figure 4:**
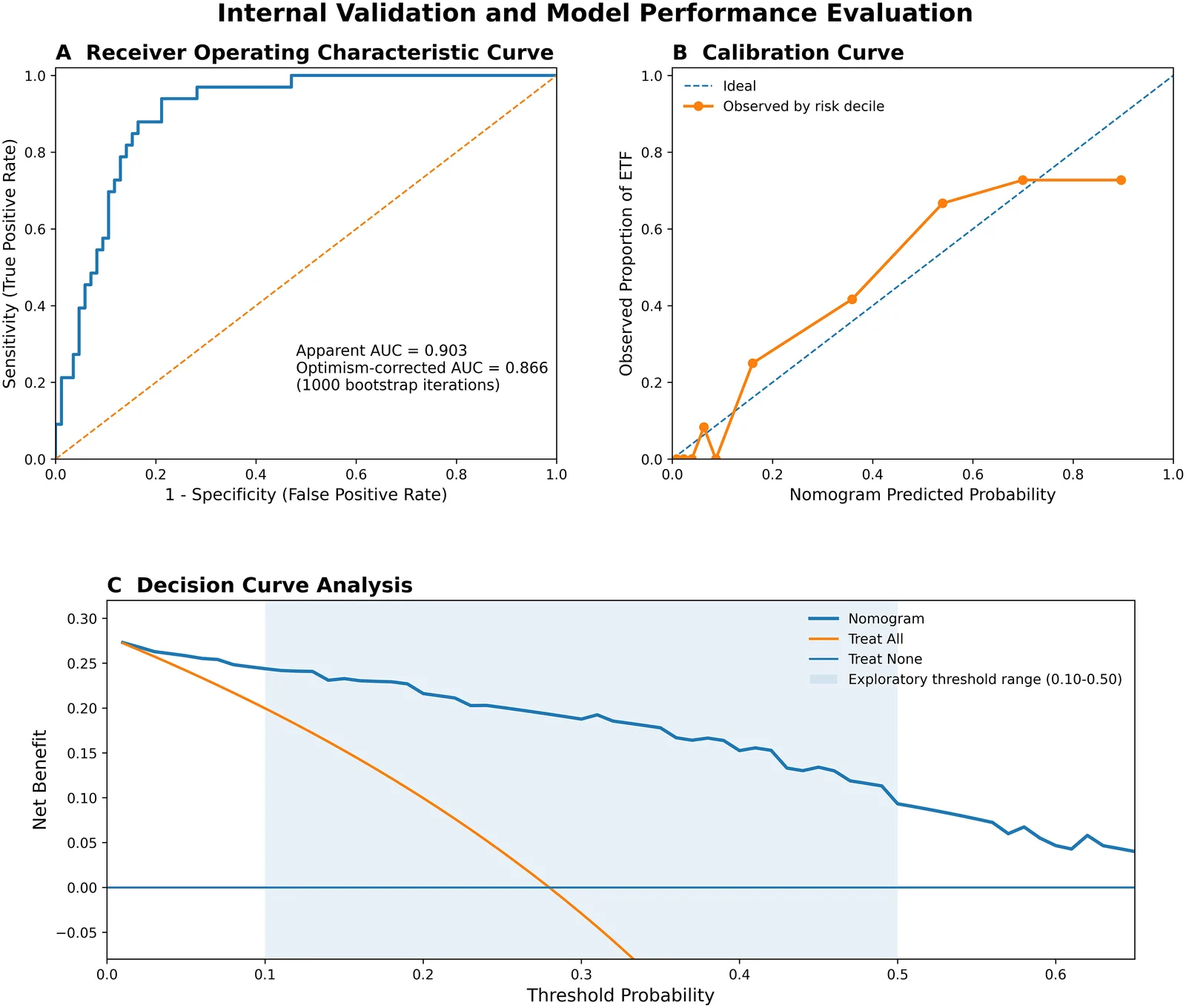
Internal model performance. **(A)** Receiver operating characteristic curve for the final nomogram; the apparent ROC-AUC was 0.903 and the optimism-corrected ROC-AUC after 1000 bootstrap iterations was 0.866. **(B)** Calibration plot comparing predicted and observed ETF risk in the development cohort. **(C)** Decision curve analysis. The shaded 0.10-0.50 interval is labeled as an exploratory threshold range; it describes the portion of the curve in which the nomogram showed higher net benefit than the treat-all and treat-none strategies in this cohort and should not be interpreted as a validated threshold for treatment modification.

### 3.5 Clinical utility evaluated by decision curve analysis

Decision curve analysis (**Figure 4**) showed higher net benefit for the nomogram than the treat-all and treat-none strategies over an exploratory threshold range of approximately 0.10-0.50 in the development cohort. This interval is presented only to describe the decision-analytic curve and is not a validated threshold for treatment modification. The model estimates ETF risk among patients receiving the triple regimen; it does not establish that patients above or below any predicted-risk threshold would benefit from a different treatment strategy.

## 4 Discussion

To the best of our knowledge, this is among the first studies to develop and internally validate a nomogram for predicting Early Treatment Failure (ETF) in advanced HCC patients treated with SBRT, lenvatinib, and tislelizumab. Building on prior analyses that have linked baseline inflammation, hepatic reserve, and tumor burden to outcomes of systemic therapy in HCC, our study integrates these dimensions into a single quantitative instrument that may be used to estimate individualized ETF risk prior to initiating the triple regimen.

The synergy between SBRT, anti-angiogenic TKIs, and PD-1 inhibitors theoretically represents a potential complementary approach in HCC (44–47). However, the observed 27.9% ETF rate in our cohort indicates that more than one in four patients still experienced primary treatment failure despite maximal triple therapy within 6 months. This emphasizes the clinical need for pretreatment risk stratification so that high-risk patients can be spared from exposure to ineffective and costly interventions, while low-risk patients can proceed with the planned regimen.

Beyond local tumor characteristics, systemic host factors critically dictated the therapeutic response. The Systemic Immune-Inflammation Index (SII), a composite biomarker integrating platelets, neutrophils, and lymphocytes, has been associated with adverse survival outcomes in HCC patients receiving immunotherapy or targeted therapy (48, 49). In our cohort, each 100-unit increase in SII was associated with an 18% increase in the odds of ETF, suggesting that a heightened pro-inflammatory state may compromise the immune-mediated component of the treatment response (50). The mechanistic interpretation of SII in this study remains observational; we make no claim that SII is a causal determinant of treatment failure, only that its baseline level captures prognostic information.

Similarly, the Albumin-Bilirubin (ALBI) score is an objective indicator of hepatic functional reserve (35). Higher ALBI scores have been associated with impaired liver function and adverse outcomes across HCC treatments (51). In the present observational cohort, higher baseline ALBI score was associated with ETF risk; this association is prognostic and should not be interpreted as evidence that ALBI directly causes treatment failure.

The modest sample size remains an important methodological limitation. LASSO with 10-fold cross-validation was used to reduce the risk of overfitting, but it cannot eliminate selection instability in a cohort with 33 ETF events and five predictors (EPV = 6.6) (52–54). Bootstrap internal validation was therefore used to estimate optimism in the complete modeling procedure, and the optimism-corrected ROC-AUC was 0.866. The λ.1se sensitivity analysis retained four of the five λ.min predictors, with SII dropping under the stronger penalty (**Table 2**), indicating that the core predictor set was relatively stable but not invariant to penalty choice. The spline analyses did not show significant evidence of non-linearity (**Table 4**). These internal analyses strengthen transparency but do not replace external validation (30).

The nomogram may be useful for pretreatment risk stratification research, but its role in treatment selection has not been established. DCA suggested net benefit over an exploratory threshold range in this development cohort (41, 42); however, predicted ETF risk should not be used by itself to select an alternative regimen, withhold triple therapy, or direct treatment de-escalation. At most, a high predicted risk may identify patients who warrant closer multidisciplinary evaluation, enhanced monitoring, or prioritization for prospective validation studies. Comparative treatment benefit can only be established in appropriately designed prospective or comparative-effectiveness studies.

Several limitations should be considered. First, the retrospective single-center design introduces selection bias. Ten patients were excluded because baseline data required for the candidate-variable set were incomplete, and a reliable patient-level covariate comparison between all excluded and included patients was not available for the incomplete-data group. The final 118-patient modeling dataset was complete for the 24 candidate predictors by design (**Supplementary Table 5**); therefore, multiple imputation was not applied in the primary analysis. Exclusion of patients with incomplete records or insufficient non-death follow-up may nevertheless have introduced selection bias. Second, only 33 ETF events were available for the five-predictor model (EPV = 6.6), leaving residual risk of overfitting despite LASSO and bootstrap validation. Third, the model lacks external validation and should be viewed as exploratory and hypothesis-generating rather than as a clinical decision rule. Fourth, treatment was delivered within a single institutional protocol and treatment-level heterogeneity may still affect ETF. External multicenter validation and prospective evaluation are required before clinical implementation (55).

## 5 Conclusion

In conclusion, we developed and internally validated a preliminary LASSO-based nomogram integrating maximum tumor diameter, Vp4 PVTT, SII, ALBI score, and log10 AFP to estimate ETF probability in advanced HCC patients treated with SBRT, lenvatinib, and tislelizumab. The model showed promising internal discrimination and acceptable calibration. Sensitivity analysis under λ.1se retained four of the five primary predictors, and spline analyses supported the parsimonious linear specification of the continuous predictors. The nomogram remains exploratory and should not be used to select, withhold, or de-escalate treatment without external and prospective validation.

## Data Availability

The datasets and analysis code supporting the findings of this study are available from the corresponding author on reasonable request and subject to institutional and ethics approval. Patient-level data are not publicly posted because of privacy and data-protection requirements.

## Glossary

AASLD: American Association for the Study of Liver Diseases
AFP: Alpha-fetoprotein
ALBI: Albumin-Bilirubin
AUC: Area Under the Receiver Operating Characteristic Curve
BCLC: Barcelona Clinic Liver Cancer
BED: Biologically effective dose
CI: Confidence interval
CR: Complete response
CT: Computed tomography
DAMPs: Damage-associated molecular patterns
DCA: Decision curve analysis
Dmax: Maximum point dose to an organ
ECOG PS: Eastern Cooperative Oncology Group performance status
EHS: Extrahepatic spread
EQD2: Equivalent dose in 2-Gy fractions
ETF: Early treatment failure
FcγR: Fc gamma receptor
HBV: Hepatitis B virus
HCC: Hepatocellular carcinoma
HCV: Hepatitis C virus
HIF-1α: Hypoxia-inducible factor 1-alpha
ICI: Immune checkpoint inhibitor
irAE: Immune-related adverse event
IQR: Interquartile range
LASSO: Least Absolute Shrinkage and Selection Operator
mRECIST: modified Response Evaluation Criteria in Solid Tumors
MRI: Magnetic resonance imaging
MTT: Molecular targeted therapy
NLR: Neutrophil-to-lymphocyte ratio
OR: Odds ratio
ORR: Objective response rate
PD: Progressive disease
PD-1: Programmed cell death protein 1
PD-L1: Programmed death-ligand 1
PIVKA-II: Protein induced by vitamin K absence or antagonist-II
PLR: Platelet-to-lymphocyte ratio
PR: Partial response
PTV: Planning target volume
PVTT: Portal vein tumor thrombosis
Q3W: Every three weeks
RCS: Restricted cubic splines
RILI: Radiation-induced liver injury
ROC: Receiver operating characteristic
ROS: Reactive oxygen species
SBRT: Stereotactic body radiotherapy
SD: Stable disease
SII: Systemic Immune-Inflammation Index
TACE: Transarterial chemoembolization
TGF-β: Transforming growth factor-beta
TKI: Tyrosine kinase inhibitor
TRIPOD: Transparent Reporting of a multivariable prediction model for Individual Prognosis Or Diagnosis
VEGF: Vascular endothelial growth factor
V20: Volume of normal liver receiving ≥20 Gy
Vp4: Main portal trunk involvement by portal vein tumor thrombosis
