# Supplementary material for: LASSO-based nomogram to predict early treatment failure in advanced hepatocellular carcinoma treated with SBRT, lenvatinib, and tislelizumab
Source: Front Oncol. 2026 Oct 7;16:1905052. doi: 10.3389/fonc.2026.1905052 (PMC13682956; doi:10.3389/fonc.2026.1905052)
Supplement: Supplementary file 1 [file Table1.docx]

**Supplementary Material - Tables S1-S5**

Manuscript ID: 1905052 | Frontiers in Oncology

**Supplementary Table S1. Comprehensive list of 24 candidate baseline variables and their corresponding LASSO coefficients (lambda.min = 0.046)**

| Candidate variable | Variable type in model | LASSO coefficient |
| --- | --- | --- |
| Maximum tumor diameter (cm) | Continuous | 0.412 |
| Vp4 PVTT (present) | Categorical | 1.054 |
| Baseline SII (per 100 units) | Continuous | 0.186 |
| ALBI score | Continuous | 0.725 |
| Log₁₀ AFP | Continuous | 0.312 |
| Age (years) | Continuous | 0.000 |
| Sex (male) | Categorical | 0.000 |
| Etiology (HBV) | Categorical | 0.000 |
| ECOG PS (1 vs. 0) | Categorical | 0.000 |
| Child-Pugh class (B vs. A) | Categorical | 0.000 |
| Tumor number (multiple) | Categorical | 0.000 |
| Extrahepatic spread | Categorical | 0.000 |
| Neutrophil-to-lymphocyte ratio | Continuous | 0.000 |
| Platelet-to-lymphocyte ratio | Continuous | 0.000 |
| White blood cell count | Continuous | 0.000 |
| Hemoglobin | Continuous | 0.000 |
| Platelet count | Continuous | 0.000 |
| Absolute neutrophil count | Continuous | 0.000 |
| Absolute lymphocyte count | Continuous | 0.000 |
| Total bilirubin | Continuous | 0.000 |
| Albumin | Continuous | 0.000 |
| Alanine aminotransferase | Continuous | 0.000 |
| Aspartate aminotransferase | Continuous | 0.000 |
| Log₁₀ PIVKA-II | Continuous | 0.000 |

The 24 candidate baseline variables entered into LASSO regression with 10-fold cross-validation using λ.min = 0.046 (primary working value). Predictors were standardized (z-score transformation) before LASSO regression. The LASSO path forced the coefficients of 19 less informative variables to zero, and 5 variables retained non-zero coefficients. AFP and PIVKA-II were evaluated as log₁₀-transformed variables; SII was modeled per 100-unit increase.

**Supplementary Table S2. Restricted cubic spline non-linearity tests for the continuous predictors of ETF**

| Predictor | Knots (10/35/65/90 percentile) | Linear β | Linear AIC | RCS non-linearity P | ΔAIC (RCS - Linear) | Modeling decision |
| --- | --- | --- | --- | --- | --- | --- |
| Maximum tumor diameter (cm) | 3.40, 6.34, 9.65, 14.46 | 0.154 | 144.2 | 0.412 | +2.95 | Linear retained |
| Baseline SII (per 100 units) | 2.00, 4.02, 6.85, 12.39 | 0.166 | 155.6 | 0.396 | +2.47 | Linear retained |
| ALBI score | -3.07, -2.72, -2.33, -1.87 | 2.018 | 150.4 | 0.298 | +3.11 | Linear retained |
| Log₁₀ AFP | 0.78, 1.94, 3.23, 4.28 | 0.727 | 158.3 | 0.281 | +1.01 | Linear retained |

Restricted cubic spline (RCS) tests with 4 knots placed at the 10th, 35th, 65th, and 90th percentiles of each predictor distribution. The non-linearity P-value was obtained from a likelihood-ratio test comparing a logistic regression with the predictor entered linearly against an alternative model in which the predictor was entered using RCS basis functions. Lower P-values indicate stronger evidence against the linear specification. All four continuous predictors yielded non-significant non-linear components (all P>0.05), with positive ΔAIC (worse fit) for the RCS-augmented model in every case, supporting the retention of linear coding for clinical interpretability and parsimony.

**Supplementary Table S3. λ.min vs λ.1se sensitivity analysis**

| **Lambda criterion** | **Selected variables** | **n selected** | **Interpretation** | **Internal discrimination** |  |
| --- | --- | --- | --- | --- | --- |
| λ.min (=0.046) | Maximum tumor diameter; Vp4 PVTT; SII/100; ALBI score; log10 AFP | 5 | Primary model; minimum cross-validated deviance | Reference |  |
| λ.1se | Maximum tumor diameter; Vp4 PVTT; ALBI score; log10 AFP | 4 | More parsimonious sensitivity model; SII excluded under stronger penalty | Only a small decrease relative to λ.min |  |

λ.min was used for the primary model because it minimized cross-validated deviance. Under λ.1se, four of the five primary predictors were retained and SII was excluded. The small change in internal discrimination indicates that the main tumor-burden, PVTT, liver-function, and AFP predictors were relatively stable, while SII selection was sensitive to stronger penalization.

**Supplementary Table S4. Full-pipeline bootstrap stability (LASSO repeated within each resample)**

| **Bootstrap summary** | **Result** | **Interpretation** |
| --- | --- | --- |
| Iterations | 1000 | Internal validation |
| Pipeline | Standardization + LASSO feature selection + λ selection + logistic refitting repeated within resamples | Accounts for feature-selection uncertainty in internal validation |
| Selection stability | Each of the five λ.min predictors was selected in >95% of resamples | Suggests high internal selection frequency; external stability remains unknown |
| Optimism-corrected ROC-AUC | 0.866 | Internal-validation discrimination estimate |

Internal validation used 1000 bootstrap resamples with repetition of data standardization, LASSO feature selection, λ selection, and logistic-model refitting. The resulting optimism-corrected discrimination estimate is reported as an internal-validation measure only and does not replace external validation.

Supplementary Table S5. Missingness of the 24 candidate predictors in the final analytical cohort

| **Candidate predictor** | **Missing, n** | **Missing, %** | **Handling** |
| --- | --- | --- | --- |
| Maximum tumor diameter (cm) | 0 | 0.0 | Complete in final analytical cohort |
| Vp4 PVTT (present) | 0 | 0.0 | Complete in final analytical cohort |
| Baseline SII (per 100 units) | 0 | 0.0 | Complete in final analytical cohort |
| ALBI score | 0 | 0.0 | Complete in final analytical cohort |
| Log₁₀ AFP | 0 | 0.0 | Complete in final analytical cohort |
| Age (years) | 0 | 0.0 | Complete in final analytical cohort |
| Sex (male) | 0 | 0.0 | Complete in final analytical cohort |
| Etiology (HBV) | 0 | 0.0 | Complete in final analytical cohort |
| ECOG PS (1 vs. 0) | 0 | 0.0 | Complete in final analytical cohort |
| Child-Pugh class (B vs. A) | 0 | 0.0 | Complete in final analytical cohort |
| Tumor number (multiple) | 0 | 0.0 | Complete in final analytical cohort |
| Extrahepatic spread | 0 | 0.0 | Complete in final analytical cohort |
| Neutrophil-to-lymphocyte ratio | 0 | 0.0 | Complete in final analytical cohort |
| Platelet-to-lymphocyte ratio | 0 | 0.0 | Complete in final analytical cohort |
| White blood cell count | 0 | 0.0 | Complete in final analytical cohort |
| Hemoglobin | 0 | 0.0 | Complete in final analytical cohort |
| Platelet count | 0 | 0.0 | Complete in final analytical cohort |
| Absolute neutrophil count | 0 | 0.0 | Complete in final analytical cohort |
| Absolute lymphocyte count | 0 | 0.0 | Complete in final analytical cohort |
| Total bilirubin | 0 | 0.0 | Complete in final analytical cohort |
| Albumin | 0 | 0.0 | Complete in final analytical cohort |
| Alanine aminotransferase | 0 | 0.0 | Complete in final analytical cohort |
| Aspartate aminotransferase | 0 | 0.0 | Complete in final analytical cohort |
| Log₁₀ PIVKA-II | 0 | 0.0 | Complete in final analytical cohort |

The final 118-patient analytical dataset had complete data for all 24 candidate predictors after application of the prespecified complete-case eligibility criterion. Patients excluded for incomplete baseline data are described in the study flow; because complete standardized covariate data were not available for those excluded records, a formal included-versus-excluded multivariable comparison was not performed.
