# Supplementary material for: LASSO-based nomogram to predict early treatment failure in advanced hepatocellular carcinoma treated with SBRT, lenvatinib, and tislelizumab
Source: Front Oncol. 2026 Oct 7;16:1905052. doi: 10.3389/fonc.2026.1905052 (PMC13682956; doi:10.3389/fonc.2026.1905052)
Supplement: Supplementary file 2 [file Table2.docx]

**Completed TRIPOD Checklist – Round 3**

Manuscript: LASSO-Based Nomogram to Predict Early Treatment Failure in Advanced Hepatocellular Carcinoma Treated with SBRT, Lenvatinib, and Tislelizumab

| **Section / Topic** | **Item** | **Checklist item** | **Reported in section** | **Page / Figure** | **Note** |
| --- | --- | --- | --- | --- | --- |
| Title | 1 | Identify the study as developing a prediction model | Title | Title page | Title identifies LASSO-based nomogram for early treatment failure prediction. |
| Abstract | 2 | Structured summary including target population, outcome, and statistical methods | Abstract | Abstract | Structured abstract: Background/Methods/Results/Conclusion. |
| Background and objectives | 3a | Explain the clinical context and rationale | Introduction | Page 4-5 |  |
| Background and objectives | 3b | Specify the objectives and target population | Introduction | Page 5 | ETF in advanced HCC patients receiving SBRT+lenvatinib+tislelizumab. |
| Source of data | 4a | Describe the study design and data source | Methods 2.1 | Page 6 | Retrospective single-center cohort. |
| Source of data | 4b | Specify the key dates | Methods 2.1 | Page 6 | June 2021 to June 2024. |
| Participants | 5a | Specify key elements (setting, locations, eligibility criteria) | Methods 2.1-2.2 | Page 6-7 |  |
| Participants | 5b | Describe treatments received | Methods 2.3 | Page 7 | SBRT+lenvatinib+tislelizumab protocol. |
| Outcome | 6a | Clearly define the outcome to be predicted | Methods 2.5 | Page 8 | ETF (PD by mRECIST or all-cause death within 6 months). |
| Outcome | 6b | Detail blinding if used | NA | NA | Outcome assessors were board-certified radiologists blinded to predictors; statistical analyst not blinded. |
| Predictors | 7a | Clearly define all predictors used | Methods 2.4 | Page 7-8 | 24 candidate variables listed in Supplementary Table S1. |
| Predictors | 7b | Detail how and when predictors were assessed | Methods 2.4 | Page 7-8 | Baseline within 7 days prior to treatment. |
| Sample size | 8 | Explain how the study size was arrived at | Methods 2.2 | Page 6-7 | Consecutive sampling; EPV≈6.6 acknowledged as limitation. |
| Missing data | 9 | Describe how missing data were handled | Methods 2.4 and 2.6 | Page 7-9 | Complete-case analysis primary; MI sensitivity shown in Supplementary Table S5. |
| Statistical analysis methods | 10a | Describe how predictors are handled (continuous/categorical) | Methods 2.6 | Page 9 | Continuous preserved (scaling: SII/100, log10 AFP). |
| Statistical analysis methods | 10b | Specify type of model, all model-building procedures, and validation | Methods 2.6-2.7 | Page 9-10 | LASSO-logistic, full-pipeline bootstrap, calibration, DCA. |
| Statistical analysis methods | 10c | Explain how predictors are selected during model building | Methods 2.6 | Page 9 | LASSO with 10-fold CV at λ.min. |
| Statistical analysis methods | 10d | Specify if and how interaction terms are considered | NA | NA | No interactions considered; pre-specified rationale in Discussion. |
| Statistical analysis methods | 10e | Explain how model performance is assessed | Methods 2.7 | Page 10 | C-index, calibration curve, DCA; full-pipeline optimism. |
| Statistical analysis methods | 10f | Describe validation methodology | Methods 2.7 | Page 10 | 1000-iteration full-pipeline bootstrap including LASSO within each resample. |
| Risk groups | 11 | Provide details on how risk groups are created (if applicable) | NA | NA | No risk-group categorization in current version; planned for external-validation study. |
| Participants | 13a | Describe the flow of participants | Results 3.1 | Page 11 | Flow diagram; excluded 34 with reasons. |
| Participants | 13b | Describe characteristics of participants | Results 3.1, Table 1 | Page 11 |  |
| Participants | 13c | For validation, compare with development set | NA | NA | External validation pending. |
| Model development | 14a | Number of participants and events in each analysis | Results 3.1-3.2 | Page 11-12 | n=118, events=33, EPV=6.6. |
| Model development | 14b | Number of outcomes vs predictors | Results 3.3 | Page 12 | 33 events / 5 predictors. |
| Model development | 14c | Unadjusted associations (if applicable) | Supplementary Table S1 |  | LASSO path coefficients shown. |
| Model specification | 15a | Present the full prediction model (regression coefficients and intercept) | Results 3.3, Table 2 | Page 12 | Includes intercept (-3.18) and per-variable β. |
| Model specification | 15b | Explain how to use the model | Figure 3 legend, Methods 2.7 | Page 10, 13 | Nomogram with AFP log10 transformation note. |
| Model performance | 16 | Report performance measures (C-index, calibration, decision curve) | Results 3.4-3.5, Figure 4 | Page 12-13 | AUC/C-index 0.856; bootstrap optimism-corrected 0.842; calibration curve; DCA. |
| Discussion | 17a | Limitations | Discussion | Page 17 | Retrospective, single-center, limited EPV, no external validation. |
| Discussion | 17b | Interpretation in context of prior work | Discussion | Page 15-17 |  |
| Discussion | 17c | Implications | Discussion | Page 17 | Hypothesis-generating; pre-treatment risk stratification. |
| Supplementary | 18 | Discussion of supplementary materials | Supplementary |  | TRIPOD checklist, additional tables, model code. |
| Supplementary | 19 | Discussion of limitations of validation methodology | Discussion | Page 17 | Internal bootstrap only; external validation mandated. |
| Supplementary | 20 | Discussion of clinical utility and DCA | Discussion 3.5, Discussion | Page 13, 18 | DCA threshold probabilities now contextualized in Section 2.7. |
| Funding | 21 | Funding source and role | Declarations | Page 21 | No funding. |
